# Symbiote transmission and maintenance of extra-genomic associations

**DOI:** 10.3389/fmicb.2014.00046

**Published:** 2014-02-24

**Authors:** Benjamin M. Fitzpatrick

**Affiliations:** Department of Ecology and Evolutionary Biology, University of TennesseeKnoxville, TN, USA

**Keywords:** vertical transmission, horizontal transmission, symbiosis, gene-culture, coevolution, dual-inheritance theory, interspecific disequilibrium, metagenome

## Abstract

Symbiotes can be transmitted from parents to offspring or horizontally from unrelated hosts or the environment. A key question is whether symbiote transmission is similar enough to Mendelian gene transmission to generate and maintain coevolutionary associations between host and symbiote genes. Recent papers come to opposite conclusions, with some suggesting that any horizontal transmission eliminates genetic association. These studies are hard to compare owing to arbitrary differences in modeling approach, parameter values, and assumptions about selection. I show that associations between host and symbiote genes (extra-genomic associations) can be described by the same dynamic model as conventional linkage disequilibria between genes in the same genome. Thus, covariance between host and symbiote genomes depends on population history, geographic structure, selection, and co-transmission rate, just as covariance between genes within a genome. The conclusion that horizontal transmission rapidly erodes extra-genomic associations is equivalent to the conclusion that recombination rapidly erodes associations between genes within a genome. The conclusion is correct in the absence of population structure or selection. However, population structure can maintain spatial associations between host and symbiote traits, and non-additive selection (interspecific epistasis) can generate covariances between host and symbiote genotypes. These results can also be applied to cultural or other non-genetic traits. This work contributes to a growing consensus that genomic, symbiotic, and gene-culture evolution can be analyzed under a common theoretical framework. In terms of coevolutionary potential, symbiotes can be viewed as lying on a continuum between the intimacy of genes and the indifference of casually co-occurring species.

## 1. Introduction

The view that organism phenotypes can be described in terms of a dichotomy between inherited genes and non-inherited environmental factors has been an enormously useful simplification in the development of quantitative genetics and evolutionary theory (Lynch and Walsh, 1998; Futuyma, 2009). However, several fields now present important opportunities to understand the prevalence and importance of additional influences. For example, studies of gene-culture coevolution (Feldman and Zhivotovsky, 1992; Henrich et al., 2008) and symbiosis (Bright and Bulgheresi, 2010; Gilbert et al., 2010) have emphasized the roles of factors with a mixture of horizontal and vertical transmission on development and evolution. Formation of intimate symbioses has contributed to major transitions in eukaryotic evolution and community ecology (Maynard Smith and Szathmary, 1995; Selosse et al., 2006), while gene-culture coevolution can promote speciation in learning animals (Vallin and Qvarnstrom, 2011) and is associated with the emergence of *Homo sapiens* as a global ecosystem engineer (Maynard Smith and Szathmary, 1995; Vitousek et al., 1997).

The degree to which symbiotic associations and cultural traits are passed vertically from parent to offspring vs. horizontally between unrelated individuals can be critical in determining the evolutionary trajectories of traits affecting cooperation, resource use, and the functional integration of systems (symbiotic or social) more inclusive than the individual organism. Vertical transmission is thought to promote partner fidelity feedbacks between genes (Frank, 1994). That is, when host and symbiote genes maintain a statistical association across generations, the evolution of stable mutualism is likely (Frank, 1994; Doebeli and Knowlton, 1998; Fletcher and Doebeli, 2009; Wyatt et al., 2013). When vertical transmission is perfect, the evolutionary dynamics converge on the dynamics of coevolution between genes within the same genome.

Several studies have sought to extend the tools and concepts of population genetics to analyze host-symbiote and gene-culture coevolution. For example efforts have been very successful in advancing understanding and manipulation of incompatibility-inducing endosymbiotes (Turelli, 1994; Hoffmann et al., 2011). Analyses of gene-culture coevolution have been illuminating but controversial when applied to human culture (Cavalli-Sforza and Feldman, 1981; Boyd and Richerson, 1985; Richerson and Boyd, 2005; Ackland et al., 2007; Claidiere and Andre, 2012; Houkes, 2012). Even genetic models of speciation can be extended to include interactions between host and symbiote genomes (Brucker and Bordenstein, 2012, 2013). Important technical advances include the recognition that gene-culture covariance and interspecific disequilibrium (covariance between host and symbiote genes) resemble conventional linkage disequilibrium and cytonuclear disequilibrium (Feldman and Cavalli-Sforza, 1984; Sanchez et al., 2000). Further, the notion of “generalized epistasis” (Feldman and Cavalli-Sforza, 1984; Feldman and Zhivotovsky, 1992) emphasizes the potential for non-additive interactions between genotypes and cultural traits to determine the direction of evolution.

These results have inspired a generalized concept of “non-genetic inheritance” to include symbiotes, cultural traits, and potentially other non-genetic or epigenetic traits with some possibility of both vertical and horizontal transmission (Bonduriansky and Day, 2009). Proponents of this expanded view of inheritance have generally concluded that there are important similarities between models of non-genetic inheritance and classic genetic models, and that extra-genomic traits can be significant factors in evolution (Feldman and Zhivotovsky, 1992; Jablonka and Lamb, 1998; Bonduriansky and Day, 2009; Day and Bonduriansky, 2011; Odling-Smee et al., 2013). However, attempts to extend classical population and quantitative genetics theory to accommodate non-genetic inheritance have been relatively complex, making it difficult to establish general principles (Feldman and Zhivotovsky, 1992; Santure and Spencer, 2006; Tal et al., 2010; Johannes and Colomé-Tatché, 2011; Bonduriansky, 2012). Moreover, the general importance of non-genetic traits in evolution remains debated (Jablonka and Lamb, 1998; Haig, 2007; Dickins and Dickins, 2008; Bonduriansky, 2012).

The opposing conclusions from recent theoretical studies most likely arise from differing premises, assumptions, and parameter spaces. Early work on gene-culture coevolution adapted classical population genetic models to assumptions about cultural transmission, and tended to emphasize effects of selection on cultural traits (Feldman and Cavalli-Sforza, 1984; Boyd and Richerson, 1985; Feldman and Cavalli-Sforza, 1989; Feldman and Zhivotovsky, 1992). Studies of genomic imprinting have generally used a quantitative genetic framework emphasizing phenotype distributions in families (Santure and Spencer, 2006; Tal et al., 2010; Johannes and Colomé-Tatché, 2011). Day and Bonduriansky (2011) used the Price equation to develop expressions for phenotypic change owing to selection, genetic inheritance, and non-genetic inheritance. Their approach appears to be quite general, but as a trade-off for generality, it does not immediately reveal the importance of any particular mechanism of non-genetic inheritance, nor any particular measure of coevolutionary association. In contrast, Brandvain et al. (2011) made an explicit population genetic model focused on how maternal transmission of a symbiote affects interspecific covariance (disequilibrium) in a panmictic population with no selection.

Under those conditions, Brandvain et al. (2011) showed that genetic covariances (interspecific disequilibria) between neutral organelle and symbiote alleles decay rapidly with even a little horizontal transmission in a panmictic population. They concluded that imperfect vertical transmission would leave negligible statistical signature in molecular marker data and that the potential for interactions between host and symbiote genes to affect evolution would be limited. Qualitatively, the same can be said for conventional nuclear genes or cytoplasmic and nuclear genomes; linkage disequilibria (covariances) decay rapidly in panmictic populations (Lewontin, 1974; Hartl and Clark, 1997). However, conditions favoring persistent covariance between nuclear genes or between cytoplasmic and nuclear genes are common in nature (Arnold, 1993; Hewitt, 2001; Zapata et al., 2001; Laurie et al., 2007). Moreover epistasis is widely acknowledged as an important factor in genome evolution and speciation (Wolf et al., 2000; Coyne and Orr, 2004; Petkov et al., 2005; Weinreich et al., 2005; Muir and Moyle, 2009; Rohlfs et al., 2010), and Drown et al. (2013) showed conditions under which interspecific epistasis would favor an evolutionary transition from horizontal to vertical transmission in a host-symbiote system.

The apparently unresolved question is whether associations between host and symbiote genes have fundamentally different dynamics from associations between genes in the same genome (as implied by Brandvain et al., 2011), or whether fundamental dynamic similarities trump a superficial distinction between genetic and non-genetic inheritance (Day and Bonduriansky, 2011). Here, I attempt to address this question by deriving a few very simple relationships, and then illustrating some of their implications with simulations of a few biologically interesting scenarios. My conclusion might not be surprising to theoretical population geneticists; extra-genomic traits like symbiotes can be studied by extending the tools of classical population genetics (Cavalli-Sforza and Feldman, 1981; Boyd and Richerson, 1985; Sanchez et al., 2000; Day and Bonduriansky, 2011). However, my analysis might be helpful for microbial ecologists. I shed some new light on the early work on associations (disequilibria) between classical alleles and non-genetic traits (Feldman and Cavalli-Sforza, 1984) and contradict the inferred conclusions (though not the mathematical results) of Brandvain et al. (2011). My results help illustrate the value of a unified view of inheritance and coevolution (Day and Bonduriansky, 2011), with symbiosis falling on a continuum of intimacy including genes, organelles, free-living species, and even environmental factors (Lewontin, 1983; Keeling and Archibald, 2008; Odling-Smee et al., 2013).

## 2. Methods

Brandvain et al. (2011) made two claims against the evolutionary relevance of associations between host organelle and symbiote alleles (interspecific disequilibrium). First, high rates of vertical transmission of symbiotes do not result in high levels of genetic association. Second (consequently), host-symbiote interactions will not respond to natural selection on non-additive fitness effects (interspecific epistasis). The basis of these claims is a model showing rapid decay of covariances between organelle and symbiote genomes in a single randomly mating population with no selection. Therefore, I first evaluate the veracity of their result using a simpler, more traditional population genetics model.

The model follows earlier work generalizing the traditional measure of association between genes in the same genome (linkage disequilibrium) to extra-genomic associations (Feldman and Cavalli-Sforza, 1984; Feldman and Zhivotovsky, 1992), but I provide a new result, a closed-form solution for the evolutionary dynamics of extra-genomic associations. To put a finer point on the question of whether extra-genomic associations can be informative about the transmission process, I explicitly compare the dynamics of symbiote-organelle covariance with the dynamics of symbiote-nuclear covariance. To my knowledge, this has not been done previously.

Second, I evaluate the effects of population structure on patterns of extra-genomic covariance. Population structure is well known to promote within-genome covariance (linkage disequilibrium), but the impact of subdivision and gene flow on the dynamics of extra-genomic traits has rarely been investigated (Ackland et al., 2007). I derive new analytical results for equilibrium covariance in a simple admixture model (Asmussen and Arnold, 1991) and then show numerical results for a more complicated hybrid zone model modified from Dakin (2006).

Finally, I test whether epistatic interactions between host and symbiote genes can affect how a population responds to selection. I illustrate the earlier results of Feldman and Cavalli-Sforza (1989), which demonstrate an evolutionary response to selection on epistatic effects between a gene and a cultural trait. I show conditions under which host-symbiote genetic covariance is built up by selection despite the tendency for horizontal transmission to reduce covariance. I also evaluate effects of hitchhiking between host and symbiote genes in a single population and across a hybrid zone.

## 3. Results

### 3.1. Covariance between symbiote and organelle genomes

To derive the expected covariance between organelle (cytoplasmic) and symbiote genomes, consider a system where each genome has two types (haplotypes or haplotype groups): *C* and *c* for the cytoplasmic organelle, and *S* and *s* for the symbiote. If *x*_*ij*_ is the frequency of individuals with cytotype *i* and symbiote *j*, and *p*_*i*_ and *p*_*j*_ are the marginal frequencies of cytotype *i* and symbiote *j*, the covariance (cyto-symbiote disequilibrium, *D*) is

(1) $$D=x_{CS}-p_{C}p_{S}.$$

Equation (1) is the covariance between two binary random variables, and can be applied to phenotypes, genes, symbiotic states or any pair of binary categorical variables (Clark, 1984; Feldman and Cavalli-Sforza, 1984). Statistical association or disequilibrium can also be expressed as a correlation $\left(r=D/\sqrt{p_{S}p_{s}p_{C}p_{c}}\right)$. If the probability of vertical (maternal) transmission of the symbiote is *v*, the set of frequencies $\vec{x}=(x_{CS},x_{Cs},x_{cS},x_{cs})$ is expected to change across generations according to the transition matrix in Table 1. Therefore, the expected cyto-symbiote covariance in the next generation is

(2) $$\begin{array}{l} D^{\prime }={x^{\prime }}_{CS}-p_{C}p_{S}\\ \;=x_{CS}v+\left(x_{CS}+x_{Cs}\right)\left(1-v\right)p_{S}-p_{C}p_{S} \end{array}$$

Substitute *p*_*C*_ = *x*_*CS*_+*x*_*Cs*_

$$\begin{array}{l} D^{\prime }=x_{CS}v-p_{C}p_{S}v\\ \;=Dv \end{array}$$

And the general solution is *D*_*t*_ = *D*_0_*v*^*t*^. This novel result is directly comparable to the classic result for covariance between nuclear genes *D*_*t*_ = *D*_0_*c*^*t*^, where *c* is the probability of cosegregation (1−recombination rate) (Lewontin, 1974).

**Table 1:** **Transition matrix for joint symbiote and organelle genotypes**.

		***t* + 1**			
		***x*′_*CS*_**	***x*′_*Cs*_**	***x*′_*cS*_**	***x*′_*cs*_**
	*x*_*CS*_	*v* + (1 − *v*)*p*_*S*_	(1 − *v*)*p*_*s*_	0	0
*t*	*x*_*Cs*_	(1 − *v*)*p*_*S*_	*v* + (1 − *v*)*p*_*S*_	0	0
	*x*_*cS*_	0	0	*v* + (1 − *v*)*p*_*S*_	(1 − *v*)*p*_*S*_
	*x*_*cs*_	0	0	(1 − *v*)*p*_*S*_	*v* + (1 − *v*)*p*_*S*_

If the symbiosis is not obligate and exclusive, i.e., if a host can have zero symbiotes or more than one symbiote genotype, the result is similar. Let *x*_*CS*_ be the frequency of individuals with cytotype *C* and symbiote genotype *S*, regardless of what other symbiote genotypes are present. Then *x*′_*CS*_ = *x*_*CS*_*v*(1 − *h*_*S*_) + *p*_*C*_*h*_*S*_, where *h*_*S*_ is the probability of gaining a symbiote with genotype *S* by horizontal transfer (from the environment or another host). Let *D* = *x*_*CS*_ − *p*_*C*_*p*_*S*_, where *p*_*S*_ is the fraction of hosts with symbiote *S* (rather than the fraction of symbiotes with genotype *S*). In this case

(3) $$D_{t}=D_{0}\text{​}{\left[v\left(1-h_{S}\right)\right]}^{t}.$$

That is, when the ecological relationship between host and symbiote is facultative, the effective recombination rate is 1 − *v*(1 − *h*_*s*_), which is greater than the obligate case (1 − *v*).

### 3.2. Covariance between symbiote and nuclear genes

For the covariance between a symbiote genotype and an allele at a nuclear locus (with alleles *A* and *a*), the nuclear-symbiote covariance is *D* = *x*_AAS_ + $\frac{1}{2}$*x*_AaS_ − *p*_*A*_*p*_*S*_ (Asmussen et al., 1987). The relevant host-symbiote genotype frequencies in the next generation are given by

$${x^{\prime }}_{\text{AAS}}=v\left(x_{\text{AAS}}+\frac{1}{2}x_{\text{AaS}}\right)p_{A}+\left(1-v\right)p_{S}\left(x_{AA}+\frac{1}{2}x_{Aa}\right)p_{A}$$

and

$$\begin{array}{l} {x^{\prime }}_{\text{AaS}}=v\left(x_{\text{AAS}}p_{a}+\frac{1}{2}x_{\text{AaS}}+x_{\text{aaS}}p_{A}\right)\\ \;+\left(1-v\right)p_{S}\left(x_{AA}p_{a}+\frac{1}{2}x_{Aa}+x_{aa}p_{A}\right) \end{array}$$

The nuclear-symbiote covariance in the next generation *D*′ = *x*′_AAS_ + $\frac{1}{2}$*x*′_AaS_ − *p*_*A*_*p*_*S*_ simplifies to

(4) $$D^{\prime }=D\frac{1}{2}v$$

And the general solution is $D_{t}=D_{0}{\left(\frac{1}{2}v\right)}^{t}$. A pertinent special case is perfect maternal transmission (*v* = 1), which eliminates the distinction between symbiote and organelle. Covariance between a maternally transmitted organelle and a nuclear gene (cyto-nuclear disequilibrium) decays by one half each generation $\left(D_{t}=D_{0}{\left(\frac{1}{2}\right)}^{t}\right)$, just like covariance between unlinked nuclear genes (Asmussen et al., 1987).

**Figure 1:**
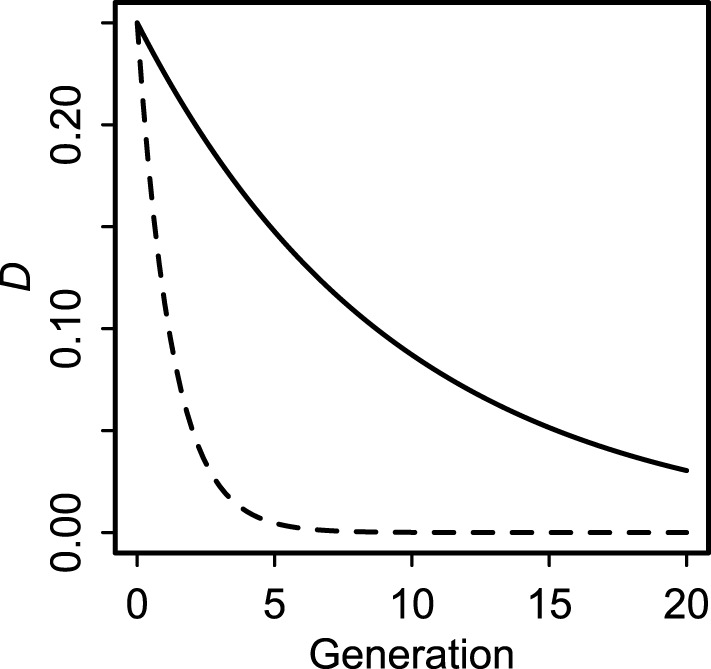
**Decay of cyto-symbiote covariance (solid line, Equation 2) and nuclear-symbiote covariance (dashed line, Equation 4) for vertical transmission probability ***v*** = 0.90**.

Even though all covariances decay rapidly in a panmictic population (Lewontin, 1974; Sanchez et al., 2000; Brandvain et al., 2011), cyto-symbiote covariance (Equation 2) decays much more slowly (by a factor of 2) than nuclear-symbiote covariance (Equation 4) whenever there is non-zero maternal transmission (Figure 1). The key difference between host-symbiote transmission and conventional gene or organelle transmission is that completely free recombination corresponds to a cosegregation probability of $\frac{1}{2}$, whereas completely horizontal transmission corresponds to a vertical transmission probability of zero. Thus, although conventional linkage disequilibrium between unlinked loci is expected to decay no faster than by $\frac{1}{2}$ each generation, host symbiote covariance can drop to zero in one generation if there is no tendency for parent-offspring transmission. Aside from this quantitative difference, the population genetic principles describing the relationship between nuclear and cytoplasmic genomes (Asmussen et al., 1987; Arnold et al., 1988; Arnold, 1993) can be extended to the relationship between hosts and symbiotes. This insight underscores a fundamental continuity between nuclear genes, organelles, and symbiotes.

### 3.3. Host-symbiote covariance in structured populations

#### 3.3.1. Continent-island structure

**Figure 2:**
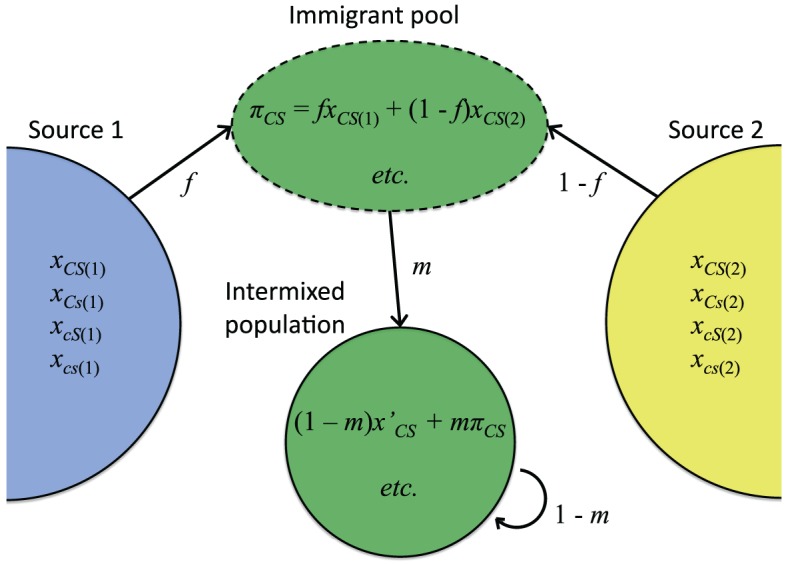
**Intermixture model used to illustrate how covariance between a cytoplasmic genotype ***C*** and symbiote genotype ***S*** can be maintained by gene flow (Equations 5–7)**. Relative contribution of source population 1 is represented by *f*. *x*_*CS*(*i*)_ is the frequency of individuals with cytoplasmic genotype *C* and symbiote genotype *S* in Source *i*, and π_*CS*_ is the frequency of that genotypic combination in the immigrant pool. *x*′_*CS*_ is the frequency of that genotypic combination in native-born residents in the intermixed population, and *m* is the immigration rate.

Admixture or immigration between populations with divergent allele frequencies is a common cause of within-genome association (conventional linkage disequilibrium) in nature (Conner and Hartl, 2004). To assess the effect of this kind of population structure on host-symbiote genetic associations, first consider a simple continent-island admixture model (Asmussen and Arnold, 1991) in which a focal population receives a proportion *m* of immigrants from two or more divergent source populations (Figure 2). Let the frequency of immigrants with cytoplasmic allele *C* and symbiote *S* be π_*CS*_. After immigration, the covariance between cytoplasmic and symbiote genotypes (cyto-symbiote covariance *D*_*CS*_) is

(5) $$\begin{array}{l} {D^{\prime }}_{CS}=\left(1-m\right){x^{\prime }}_{CS}+m\pi_{CS}-{\bar{p}}_{C}{\bar{p}}_{S}\\ \;=v\left(1-m\right)D_{CS}+mD_{m}+\text{cov}(p_{C},p_{S}) \end{array}$$

where *D*_*m*_ is the covariance among immigrants. The averages (*p*_*C*_ and *p*_*S*_) and covariance of the allele frequencies are over immigrant and resident born sets with weights *m* and 1 − *m*. Exactly the same dynamics can be derived for conventional nuclear or cyto-nuclear linkage disequilibria (Asmussen and Arnold, 1991). If we assume the source populations are themselves unchanged by gene flow, so that *D*_*m*_ and the allele frequencies are constant, this difference equation has a non-zero equilibrium at

(6) $${\hat{D}}_{CS}=\frac{mD_{m}}{1-v\text{​}\left(1-m\right)}.$$

Likewise for nuclear-symbiote covariance,

(7) $${\hat{D}}_{NS}=\frac{mD_{m}}{1-\frac{1}{2}v\text{​}\left(1-m\right)}.$$

Setting *v* = 1 in Equation (7) recovers Asmussen and Arnold's (1991) solution for cyto-nuclear disequilibrium. Thus, interspecific genetic covariances can be maintained by immigration. Moreover, ${\hat{D}}_{CS}>{\hat{D}}_{NS}$; the expected cyto-symbiote covariance is greater than the expected nuclear-symbiote covariance at equilibrium between immigration and recombination.

#### 3.3.2. Stepping-stone structure

To investigate the effects of more complicated population structure on cyto-symbiote and nuclear-symbiote covariances, I added a symbiote to the stepping stone model analyzed thoroughly for cyto-nuclear covariance by Dakin (2006). The basic framework is a line of demes between two source populations. Dispersal occurs only between adjacent demes and mating is random within demes (Kimura and Weiss, 1964; Goodisman et al., 1998; Dakin, 2006). This framework can be modified to include some other classic models as special cases, including the continent-island (above) and two-population intermixture models (Nei and Li, 1973; Asmussen and Arnold, 1991).

First, I determined how to incorporate a symbiote with vertical transmission probability *v* into Dakin's (2006) deterministic recursion equations (Appendix Equation 9). I then iterated the system of equations to illustrate transient and equilibrium patterns. I followed Dakin (2006) by initializing ten stepping stones, the first five (nearest source 1) fixed for *A, C*, and *S*, and the second five (nearest source 2) fixed for *a, c*, and *s*. This simulates secondary contact. Each time step began with symmetrical dispersal between neighboring demes, followed by random mating within demes. In one set of simulations, I followed Dakin (2006) in assuming infinite source populations unaffected by gene flow (remaining fixed for their respective alleles for all time). In a second set, I allowed gene flow into the source populations, assuming they received half the number of immigrants (because they have only one rather than two neighbors). I report the covariances among loci in the adults (after dispersal, before mating); covariances in the offspring are lower in magnitude (by factors of *v*, $\frac{v}{2}$, and $\frac{1}{2}$) but the differences between cyto-symbiote, nuclear-symbiote, and cyto-nuclear associations are greater.

**Figure 3:**
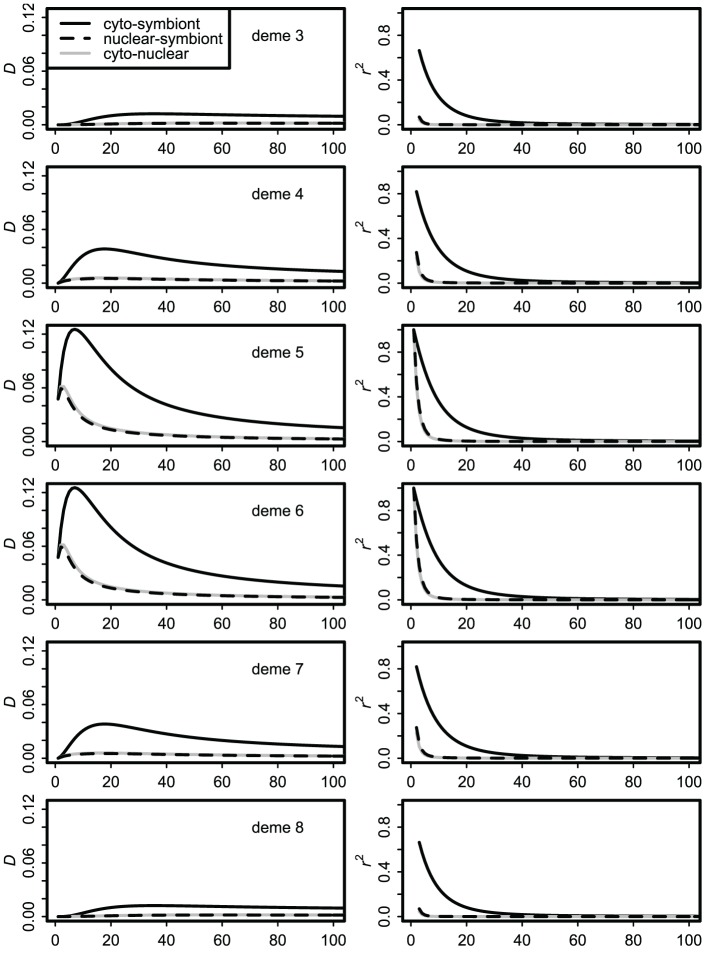
**Dynamics of covariances over the first 100 generations of secondary contact with immigration rate ***m*** = 0.10 and vertical transmission probability ***v*** = 0.90**. Only the middle six demes are shown; demes 1, 2, 9, and 10 have essentially flat lines at this scale.

Dynamics of the stepping stone model over the first 100 generations of contact are illustrated in Figure 3. Over this time frame, the difference between the infinite source and finite source models was negligible. With moderate immigration (*m* = 0.10 shown) the central demes (at the initial contact front) show transient covariances many times higher than their equilibrium values, as shown previously for cyto-nuclear covariances (Asmussen and Arnold, 1991). Most important, with high but imperfect vertical transmission (*v* = 0.90 shown), the cyto-symbiote association is greater and shows larger changes in time and space than the nuclear-symbiote or cyto-nuclear associations.

**Figure 4:**
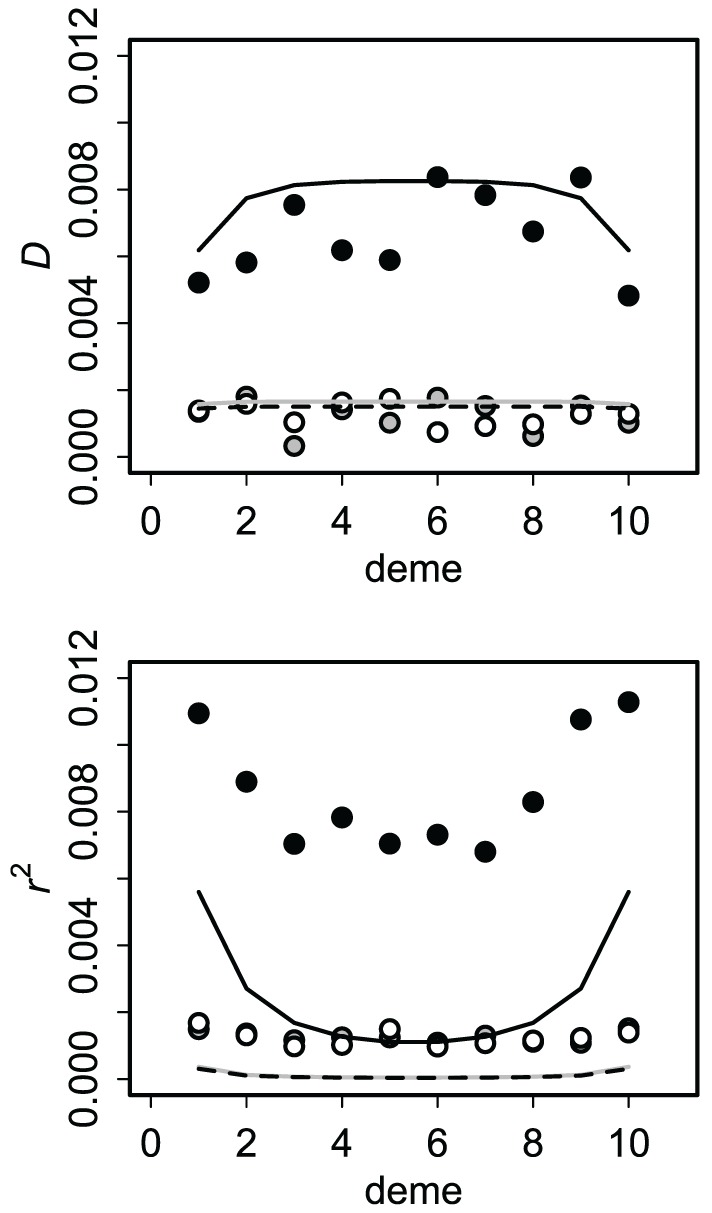
**Asymptotic covariances after 1000 generations of secondary contact with immigration rate ***m*** = 0.10 and vertical transmission probability ***v*** = 0.90**. Circles show averages of 100 replicates of the stochastic version of the model (1000 individuals per deme) and lines show the deterministic (no drift) expectations. Cyto-symbiote associations (black circles, solid line) are several times greater than nuclear-symbiote (open circles, dashed line) and cyto-nuclear (gray) associations. The correlation (*r*^2^) is affected more by drift.

Allele and genotype frequencies were very close to their asymptotic values within a few hundred generations. For the finite source model, all covariances approached zero, as expected (Asmussen and Arnold, 1991). Asymptotic results for the infinite source model (after 1000 generations) presumed to reflect equilibrium are summarized in Figure 4. Cyto-nuclear results for the allelic and genotypic covariances agree with those of Dakin (2006). Cyto-symbiote associations are many times higher, as expected from equations 2 and 4 for a high probability of vertical transmission (*v* = 0.90 shown).

To check the effects of genetic drift on these predictions, I also modeled the system with finite populations. At each generation in each deme, I sampled *N* = 1000 adults from the genotype distribution in focal deme with probability 1 − *m* and from each neighboring deme with probability *m*/2. Then I sampled *N* = 1000 offspring given the adult genotype frequencies and transmission probabilities. Results for this stochastic version were similar to the deterministic results for asymptotic covariances (Figure 4). Drift tends to increase genetic associations, but this is somewhat masked by its effects on allele frequencies. The correlation $\left(r=D/\sqrt{p_{S}p_{s}p_{C}p_{c}}\right)$ is more sensitive than the covariance (Figure 4) because *r* accounts for local allele frequencies.

### 3.4. Generalized epistasis and selection: cattle as symbiotes

Feldman and Cavalli-Sforza (1984) pointed out that the covariance between genes and a culturally transmitted trait could be described by a disequilibrium (*D* or *r*) in exactly the same way as the standard covariance between genes, and Sanchez et al. (2000) observed that gene-culture covariance and host-symbiote covariance were formally analogous to cytonuclear disequilibrium (all being covariances between binary random variables). My analysis shows that this similarity is deeper than the summary statistics; the evolutionary associations in host-symbiote and gene-culture systems follow the same kind of dynamics as Mendelian genetic systems. It follows that evolutionary relationships between host and symbiote genes should respond to epistatic selection (contra Brandvain et al., 2011). That is, the notion of epistasis can be generalized to include interactions between traits with different inheritance mechanisms (Feldman and Zhivotovsky, 1992), just as the notion of coevolution can be extended to include interactions between genes in the same genome (Lovell and Robertson, 2010).

To underscore this fundamental correspondence, consider the coevolutionary model of human lactose absorption and dairy farming investigated by Feldman and Cavalli-Sforza (1989). Their model included an autosomal (diploid) gene affecting lactase activity, and milk use as a binary cultural trait. This is a model of interspecific “generalized epistasis” because the fitness effect of a genotype depends on the presence or absence of the interaction between humans and cattle (Feldman and Cavalli-Sforza, 1989; Feldman and Zhivotovsky, 1992). The change in frequency of the absorption allele depended strongly on the degree of vertical transmission of the cultural trait (the probability that children of milk users would become milk users). The model applies equally well if we characterize dairy farming as a symbiotic association between humans and cattle. If cattle or farms tended to be passed from parent to offspring, the gene-culture covariance describing an association between human genotype and behavior (milk use) is exactly the same as host-symbiote covariance describing an association between human genotype and symbiote (cattle) presence.

I implemented the Feldman and Cavalli-Sforza (FCS) model as follows (see the online appendix and Feldman and Cavalli-Sforza, 1989 for mathematical and coding details). The first step is random mating with imperfect mother-offspring transmission of the symbiote (*U* vs. *u* for present or absent) and Mendelian transmission of an autosomal diploid locus determining whether a host benefits or suffers a net fitness cost from the symbiosis (Table 2). In the second step, following Feldman and Cavalli-Sforza (1989), offspring have a chance of picking up the symbiote via horizontal transmission. That is, each individual without the symbiote has probability *fp*_*U*_ of becoming infected (*p*_*U*_ is the frequency of infection/symbiosis among other hosts in the population). The third step is selection according to Table 2, and the resulting genotypes mate at random to produce the next generation.

**Table 2:** **Fitness of host genotypes participating in the symbiosis (*U*) or not (*u*) in the Feldman and Cavalli-Sforza (FCS) model**.

	**Host genoype**		
	***AA***	***Aa***	***aa***
Infected (*U*)	1 + *s*_1_	1 + *s*_1_	1 − *s*_2_
Uninfected (*u*)	1	1	1

*If both s_1_ and s_2_ > 0, there is sign epistasis because the effect of the EGT is negative or positive depending on the host genotype*.

To illustrate that selection can operate on interspecific epistatic effects despite imperfect vertical transmission, I present numerical results for a range of vertical transmission rates given selection *s*_1_ = 0.05 and *s*_2_ = 0.15, and horizontal transmission rate *f* = 0.5. These values are among those used by Feldman and Cavalli-Sforza (1989) in their discussion of the coevolution of lactase and dairying, and I chose them by trial and error for the simple purpose of providing a counterexample to the conjecture that imperfect transmission will make interspecific epistatic effects unresponsive to selection (Brandvain et al., 2011).

**Figure 5:**
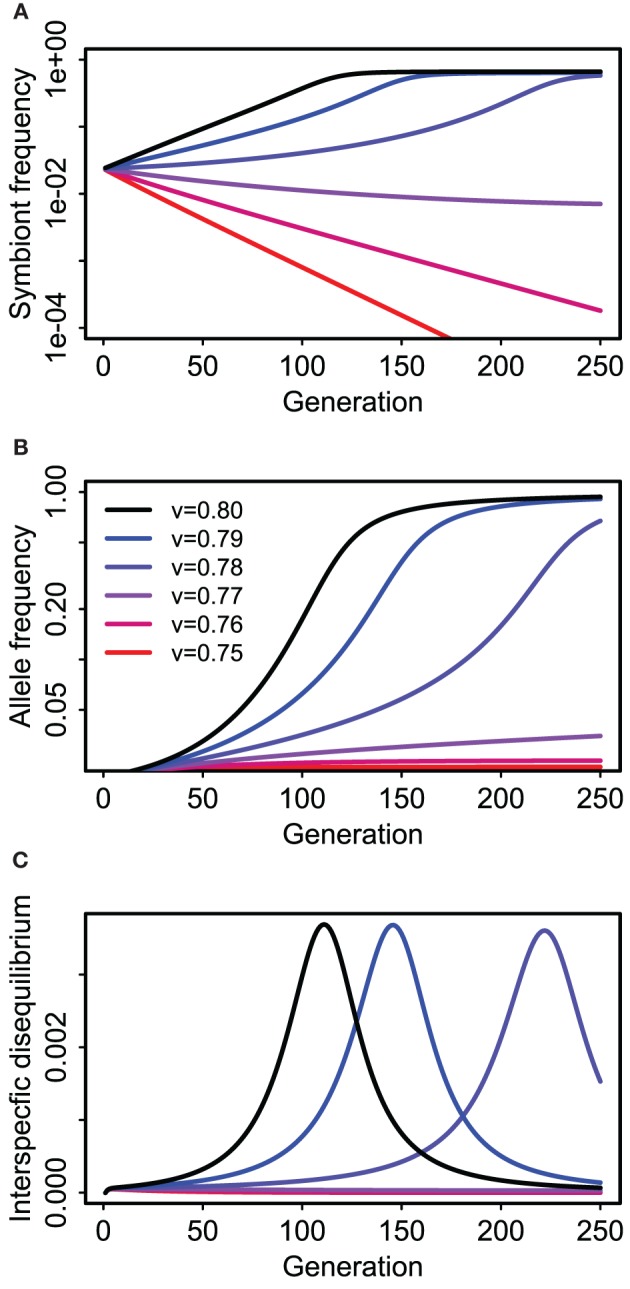
**Coevolution of a “host” gene and presence or absence of a cultural/symbiotic trait in the Feldman and Cavalli-Sforza (FCS) model for a single population**. Panel **(A)** shows prevalence of hosts (e.g., humans) with the symbiote (e.g., dairy cattle) over time for six different vertical transmission rates (see legend in **B**). Blue and black lines approach equilibrium values between 0.5 and 1.0 (Note logarithmic y-axis). **(B)** shows concurrent changes in frequency of the allele affecting fitness of hosts with and without the symbiotic relationship (e.g., a lactose absorption allele). **(C)** Shows the correlation (interspecific disequilibrium) between the host allele and symbiote presence.

Perfect vertical transmission is not required. Rather, epistatic fitness effects can drive coevolution between host and symbiote genes if the vertical transmission rate is above some threshold value determined by the strength and mode of selection and the probability of horizontal transmission (Figure 5). In the specific example depicted (Figure 5), there is a strong deleterious effect of the symbiosis (or cultural trait) on the “wild-type” genotype (*s*_2_ = 0.2) relative to its benefit for the mutant (*s*_1_ = 0.05), that is, the additive effect of the symbiote is negative when the *A* host allele is rare. Nonetheless, the symbiosis can spread (along with the *A* allele) if vertical transmission is strong enough to maintain an association between the symbiote and the host allele that makes it advantageous (*v* > 0.76 in this case, with *f* = 0.5).

Feldman and Cavalli-Sforza (1989) allowed transmission (*v* and *f*) to vary with genotype. The resulting dynamics differ only quantitatively from the simplified version used here. Day and Bonduriansky (2011) analyzed a version of the FCS model including self-learning. Individuals could (with some probability) choose the cultural practice that best suited their genotype (analogous to partner choice in symbiosis). They solved for a threshold strength of selection above which milk use increases when rare. This threshold was lowered by self-learning. High rates of vertical transmission lowered the threshold when self-learning was weak, but increased it when self-learning was strong.

### 3.5. Hitchhiking effects

To evaluate the potential for selection on extra-genomic traits to drive changes in organelle genotype frequencies via hitchhiking, I evaluated the correlated response of organelle genotype frequencies to selection in the basic model, the FCS model, and the hybrid zone model. The effect of an advantageous, maternally transmitted symbiote on a neutral cytoplasmic marker can be found for the basic model (Equation 2 and Table 1) by adding a fitness advantage of 1 + *s* for individuals with symbiote *S*. Then the frequency of the cytoplasmic allele *C* changes as

(8) $${p^{\prime }}_{C}=\frac{x_{CS}\left(1+s\right)+x_{Cs}}{\bar{W}}=p_{C}+\frac{sD}{\bar{W}}$$

where $\bar{W}=1+sp_{S}$ is mean fitness.

To evaluate hitchhiking effects of a non-obligate symbiote, I added a maternally inherited cytoplasmic marker to the FCS model. I numerically iterated the FCS model to illustrate hitchhiking effects for three kinds of conditions.

First, for comparison to the standard case of a neutral marker linked to an advantageous mutation (Maynard Smith and Haigh, 1974; Barton, 2000), I used a single panmictic population with a uniformly advantageous symbiote in the FCS framework (*s*_1_ = −*s*_2_ = *s*). The symbiote was initially in complete disequilibrium with a cytoplasmic marker. I.e., there were two kinds of individuals: *CU* and *cu*. Each *CU* produced *CU* offspring with probability *v* and *Cu* offspring with probability 1 − *v*. *Cu* and *cu* offspring were converted to *CU* and *cU* with probability *fp*_*U*_, where *p*_*U*_ was the frequency of individuals with the symbiote at that time. Individuals with the symbiote had relative fitness 1 + *s*. For comparison to the standard Maynard-Smith and Haigh (MSH) model, I calculated the expected final frequency of the cytoplasmic marker (*p*_*C*,∞_) in terms of initial frequencies of *C* and *U* and selection (*s*) from Barton (2000) Equation (1): *p*_*C*,∞_ = *p*_*C*,0_ + (1 − *p*_*C*,0_)*p*^*r*/*s*^_*U*,0_, with the approximate effective recombination rate *r* = 1 − *v* (1 − *f*) from Equation (3). This MSH expectation is not perfectly identical to the cyto-symbiote hitchhiking model (there are differences in ploidy, initial conditions, and the “effective recombination rate” is only an approximation), but the magnitude of hitchhiking should be comparable if the claim is true that the FCS model produces evolutionary dynamics for host-symbiote gene pairs that are comparable to dynamics for conventional genes.

**Figure 6:**
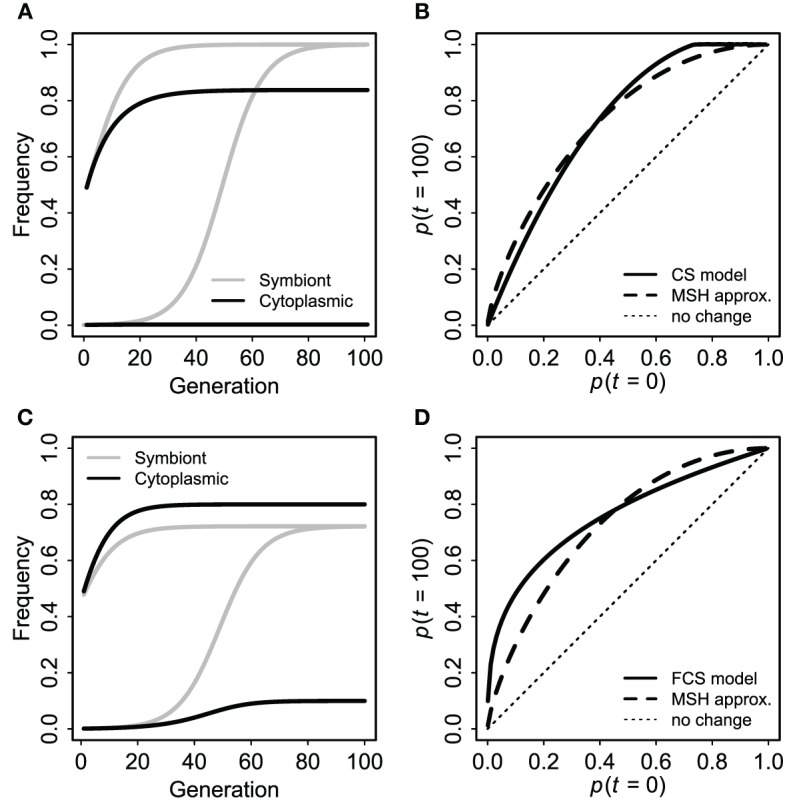
**Cyto-symbiote hitchhiking effect over 100 generations with a universally advantageous symbiote in a panmictic population**. Panel **(A)** shows dynamics of symbiote prevalence and cytoplasmic marker frequency for an initially rare symbiote (bottom pair of lines) and for 1:1 admixture (upper pair) under the basic (CS) model with selection *s* = 0.15 and vertical transmission *v* = 0.9025. **(B)** Shows final cytoplasmic marker frequencies (after 100 generations) as a function of the initial frequency (of both cytoplasmic marker and symbiote) for the basic CS model in comparison to the Maynard Smith and Haig (MSH) approximation for a pair of nuclear loci with *s* = 0.15 and *r* = 1 − *v* = 0.0975. **(C,D)** Show analogous results from the Feldman and Cavalli-Sforza (FCS) model with a universally advantageous symbiote (*s*_1_ = 0.15, *s*_2_ = −0.15), vertical transmission probability *v* = 0.95 and horizontal transmission probability *f* = 0.05. Here I used a recombination rate 1 − *v*(1 − *f*) = 0.0975 for the MSH model, making the two sets of results as comparable as possible.

As expected based on standard population genetics and Equations (2, 3, and 8), the hitchhiking effect of a spreading symbiote depends on the strength of selection, the co-transmission rate, and the initial conditions (Table 3). The examples in Figure 6 illustrate the similarity between cyto-symbiote hitchhiking (or extra-genomic hitchhiking in general) and the hitchhiking effect of one nuclear locus on another (Maynard Smith and Haigh, 1974; Barton, 2000). Graphs for other parameter combinations are available as Appendix A.

**Table 3:** **Cytoplasmic marker frequencies after 100 generations of cyto-symbiote hitchhiking in the FCS model for various vertical transmission rates (*v*) and fitness effects**.

***v***	***s* = 0.10**	**0.15**	**0.20**
*p*_0_ = 0.001			
0.90	0.006	0.038	0.095
0.95	0.039	0.100	0.164
0.99	0.082	0.151	0.215
*p*_0_ = 0.50 (admixture)			
0.90	0.711	0.777	0.818
0.95	0.752	0.805	0.838
0.99	0.775	0.821	0.849
Contact zone midpoint			
0.90	0.627	0.680	0.712
0.95	0.671	0.709	0.734
0.99	0.692	0.725	0.747
Contact zone with epistasis			
0.90	0.591	0.673	0.725
0.95	0.655	0.705	0.735
0.99	0.668	0.708	0.736

*The first case (p_0_ = 0.001) represents an initially rare symbiote in a panmictic population, and the second (p_0_ = 0.50) represents 1:1 admixture (the cytoplasmic marker was initially perfectly associated with the advantageous symbiote). The third and fourth cases represent contact zones (mean allele frequencies of demes 50 and 51 out of 100 total stepping stones). In the first three cases, the symbiote is universally advantageous (*s* = *s*_1_ = − *s*_2_). In the final, epistatic case *s*_2_ = $\frac{1}{2}$*s*_1_*.

Second, to evaluate the dynamics of secondary contact with a universally advantageous symbiote, I used the stepping-stone model described above with inheritance and selection according to the FCS model after dispersal. I used 100 stepping stones and iterated the deterministic difference equations for 100 generations after secondary contact for several combinations of transmission rates (*v* and *f*) and selection strengths (*s*_1_ = −*s*_2_ = *s*).

Finally, I repeated the simulations of secondary contact with selection according to the FCS model with sign epistasis (Weinreich et al., 2005). That is the effect of the symbiote is positive or negative according the host genotype, as in the original formulation (Feldman and Cavalli-Sforza, 1989). For the examples depicted, I varied the strength of selection but kept the ratio constant (*s*_2_ = *s*_1_/2).

**Figure 7:**
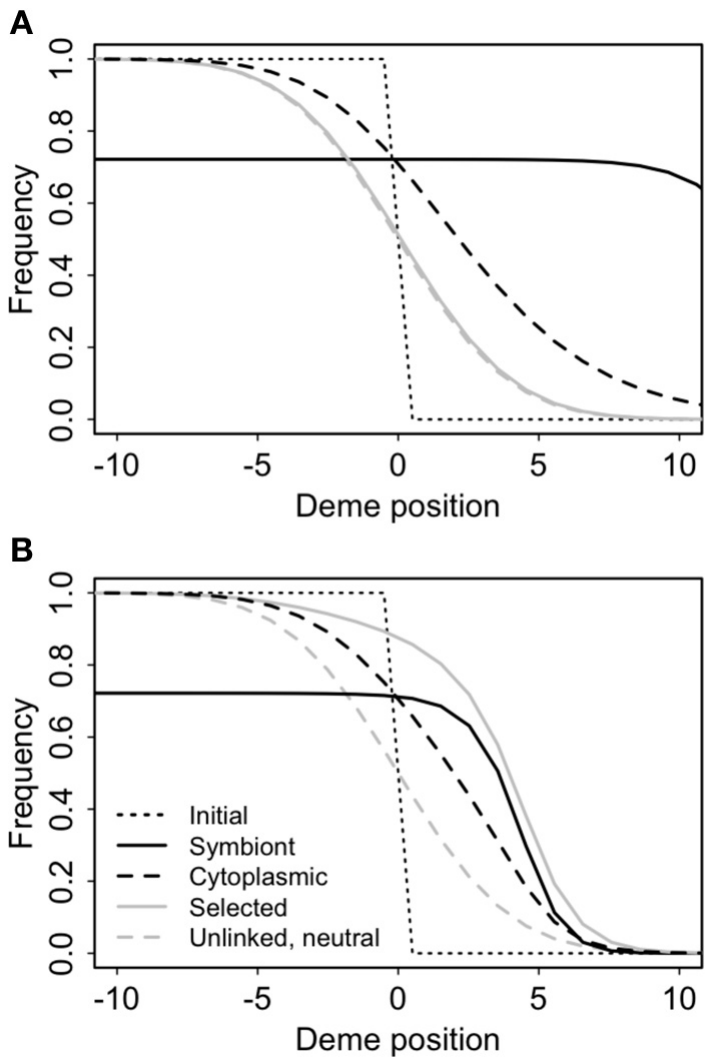
**Cyto-symbiote hitchhiking effect after 100 generations of the Feldman and Cavalli-Sforza (FCS) model in a secondary contact zone with 100 demes in a stepping stone pattern (only the central 20 demes are shown)**. Selection favoring the symbiote was *s*_1_ = 0.15, vertical transmission probability *v* = 0.95 and horizontal transmission probability *f* = 0.05. Panel **(A)** shows results for a universally advantageous symbiote with no epistasis (*s*_2_ = −*s*_1_). **(B)** Shows results for sign epistasis with *s*_2_ = $\frac{1}{2}$*s*_1_.

Cyto-symbiote hitchhiking can cause significant differential introgression of cytoplasmic DNA (Figure 7, Table 3). The spatial cline for the allele frequency of a neutral nuclear marker is unaffected by the spread of a symbiote across a hybrid zone, but there is a significant spatial displacement of the cline for a neutral cytoplasmic marker owing to the slow decay of cyto-symbiote covariance. As it does for intragenomic hitchhiking (Barton, 2000), spatial structure reduces the overall hitchhiking effect (Table 3) by slowing the response to selection and allowing more time for recombination. Introgression is generally slower with epistasis (Table 3), in part because the overall selective advantage of the symbiote is reduced when *s*_2_ > −*s*_1_ (Table 3), and in part because the advantage of the symbiote depends on co-introgression of the epistatic nuclear allele, producing a steeper, more slowly advancing wave front (Figure 7).

## 4. Discussion

The simple models presented here extend previous theoretical work on nuclear, cyto-nuclear, and extra-genomic covariances (Nei and Li, 1973; Asmussen and Arnold, 1991; Feldman and Zhivotovsky, 1992; Goodisman et al., 1998; Sanchez et al., 2000; Dakin, 2006; Brandvain et al., 2011). My results illustrate a very important conceptual and mathematical correspondence between the dynamics of associations between genes in different genomes and associations between genes in the same genome. Although there have been doubts about the stability of coevolutionary associations between host and symbiote genes with less than 100% vertical transmission (Brandvain et al., 2011), such concerns are no more or less valid for extra-genomic associations than for associations between genes within a genome. This inference can be extracted from the general frameworks of dual-inheritance theory (Cavalli-Sforza and Feldman, 1981; Feldman and Cavalli-Sforza, 1984; Boyd and Richerson, 1985; Feldman and Zhivotovsky, 1992) or non-genetic inheritance (Bonduriansky and Day, 2009; Day and Bonduriansky, 2011). My analysis uses more specific models to put a finer point on the idea that coevolution between genomes and within genomes share fundamental similarities.

The study of covariance between genes in symbiotic species is important because assortment between mutually beneficial genotypes is a key to the evolutionary origin and stability of mutualism (Frank, 1994; Fletcher and Doebeli, 2009; Wyatt et al., 2013). Vertical transmission does not guarantee the evolution of mutualism; persistent conflicts are sometimes evident within genomes or between host and organelle or endosymbiote genomes (Burt and Trivers, 2006). However, cross-generation covariance between host and symbiote genes can strongly affect the direction of evolution.

My analysis shows that extra-genomic covariances follow the same dynamics as conventional linkage disequilibria. In situations where conventional linkage disequilibria tend to persist (e.g., structured populations), extra-genomic covariances can also be expected if there is some level of vertical transmission. Therefore, extra-genomic covariances might often be informative about rates of vertical vs. horizontal transmission. In addition, the potential for non-additive fitness effects to generate significant evolutionary change at the level of symbiotic or cultural systems can be similar to the potential for epistasis to affect change at the level of conventional genomes.

### 4.1. Similarities and differences between symbiote transmission, cultural transmission, and gene transmission

The derivations of Equations (2–7) expose a striking dynamic similarity of within-genome and between-genome covariances. Disequilibria (covariances) are expected to decay as exponential functions of time. The consequence in a large, panmictic population is that we expect covariances to become negligible after a few generations (Lewontin, 1974; Sanchez et al., 2000; Brandvain et al., 2011). However, even in such an idealized population, the rate of decay depends on the probability of co-transmission. Therefore, maternally transmitted symbiotes will tend to have higher covariance with maternally transmitted genes (e.g., cytoplasmic genomes) than with nuclear genes (Figure 1).

Two differences between intra-genomic and extra-genomic associations can modify the exponential decay pattern. First, zero linkage corresponds to a cotransmission probability of 0.5 for a pair genes within a genome, whereas zero vertical transmission corresponds to a cotransmission probability of 0.0 for host and symbiote genes. This means the decay of extra-genomic covariance can be instantaneous (when *v* = 0). Second, if the symbiotic relationship is not obligate, some offspring can be without the symbiote (i.e., they neither inherited it from a parent nor picked it up from someone else or the environment). This effectively increases the decay of extra-genomic covariance and causes a systematic tendency for the symbiosis to be lost in the absence of selection or an environmental source (Feldman and Cavalli-Sforza, 1989; Lipsitch et al., 1995; Sanchez et al., 2000).

### 4.2. Using extra-genomic covariance to make inferences about vertical transmission

Given the rapid decay of covariances in panmictic populations, even for physically linked genes, lack of evident covariances cannot be interpreted as evidence against vertical transmission (Lewontin, 1974; Sanchez et al., 2000; Brandvain et al., 2011). However, when present, covariances can be informative. My analysis shows how even imperfect maternal transmission of symbiotes generates greater cyto-symbiote association than cytonuclear or nuclear-symbiote association (Figure 1). This result suggests that extra-genomic covariance can be used to make inferences about rates of vertical transmission in structured populations. Cytonuclear covariance and covariance between nuclear genes with known linkage relationships can be used as benchmarks for the detectability of vertical transmission of symbiotes. In particular, maternally inherited symbiotes are expected to have greater covariance with maternally inherited genes than with biparentally inherited genes (by a factor of 2^*t*^ in the simple case of neutral decay in a panmictic population). Maternal transmission of bacteria might be common in animals (Funkhouser and Bordenstein, 2013). Nuclear and cytonuclear covariances are frequently observed in nature, particularly in zones of admixture or hybridization (Arnold, 1993; Hewitt, 2001; Zapata et al., 2001; Laurie et al., 2007). Interspecific covariances should be common in those same situations for symbiotes with substantial vertical transmission.

Recently, Thierry et al. (2011) documented extra-genomic covariance between whitefly genotypes and prevalence of several endosymbiotic bacteria in a zone of recent contact between native and introduced whiteflies. These endosymbiotes are maternally transmitted and showed strong correspondence between mtDNA and symbiotypes in hybrids (Thierry et al., 2011). However, the symbiotes have varying degrees of horizontal transmission and only one of three is obligate (Ahmed et al., 2013). Despite imperfect vertical transmission, host mtDNA and symbiote genotypes covary in the admixture zone (Thierry et al., 2011) and at a regional scale (Ahmed et al., 2013). These observations from nature indicate that it will be feasible to test for patterns of extra-genomic covariance consistent with maternal transmission in many wild systems. In the whitefly system, it will be interesting to compare patterns of nuclear gene flow with those of mtDNA, primary endosymbiotes, and secondary endosymbiotes.

### 4.3. Implications for symbiosis and extra-genomic coevolution

Selection on non-genetic traits such as symbioses or learned behaviors can produce hitchhiking effects and extra-genomic covariances even when vertical transmission is less than perfect. Even non-additive interactions (generalized epistasis) between genes in different genomes can affect the direction of evolution. This has been shown before, particularly in models of gene-culture evolution (Feldman and Cavalli-Sforza, 1989; Ackland et al., 2007; Day and Bonduriansky, 2011), but the continuity of coevolution within genomes, between genomes, and even between genes and culture is underappreciated (Brandvain et al., 2011).

All forms of coevolution depend on consistent associations between partners to produce reciprocal responses to selection, but intimate symbioses are often thought of as special. Recent enthusiasm over the evolutionary ecology of human-microbe interactions has raised broad questions about whether plants and animals should be conceptualized as “holobionts” or “meta-organisms”, including hosts and associated microbial communities as units or levels of organization in evolution and ecology (Rosenberg et al., 2007; Doolittle and Zhaxybayeva, 2010). The answer depends, in part, on whether coevolutionary dynamics between genes in different genomes (host and microbe) are similar to dynamics between genes in the same genome. The decay of associations between genomes in a panmictic population with imperfect vertical transmission might be interpreted as leaving little opportunity for non-additive interactions between genomes to drive evolution (Brandvain et al., 2011). However, my analysis and several previous studies illustrate how associations between genomes are affected by the same factors and follow the same kinds of dynamics as associations between genes within a genome. Population structure can generate and maintain associations that might affect local coevolution (Doebeli and Knowlton, 1998; Thompson, 2005; Wyatt et al., 2013) and generalized epistasis can generate associations and determine the outcome of coevolution (Feldman and Cavalli-Sforza, 1984; Feldman and Zhivotovsky, 1992; Drown et al., 2013). Interspecific epistasis can even contribute to host speciation (Brucker and Bordenstein, 2012), for example by impacting mating behavior (Sharon et al., 2010) or hybrid viability (Brucker and Bordenstein, 2013). It does not necessarily follow that host-symbiote systems should be conceptualized as “meta-organisms,” but the theoretical continuity between coevolution of genes within genomes and between genomes is encouraging for further synthesis between evolutionary genetics and evolutionary ecology.

## Author contributions

The sole author (Benjamin M. Fitzpatrick) is responsible for all aspects of this article.

## Funding

University of Tennessee.

### Conflict of interest statement

The author declares that the research was conducted in the absence of any commercial or financial relationships that could be construed as a potential conflict of interest.

## Supplementary material

The Online Appendices B–E: the R codes for this article are available in Data Sheet 1 can be found online at: http://www.frontiersin.org/journal/10.3389/fmicb.2014.00046/abstract

## Appendix

### Appendix A: expanded description of methods for simulating extra-genomic trait transmission

#### Host-symbiont covariance in structured populations

To investigate the effects of population structure on cyto-symbiont and nuclear-symbiont disequilibria, I added a symbiont to the stepping stone model analyzed for cyto-nuclear disequilibria by Dakin (2006). Within each deme, random mating and inheritance of the nuclear locus *A*, maternally transmitted cytoplasmic locus *C* and imperfectly transmitted symbiont *S* follows the set of deterministic recursions given in Equation (9).

The expected frequencies after dispersal are given by the weighted averages of adjacent demes such that a fraction *m* of each deme is composed of immigrants coming in equal proportions from the two adjacent demes (Kimura and Weiss, 1964; Goodisman et al., 1998; Dakin, 2006). I followed Dakin (2006) by initializing ten stepping stones, the first five (nearest source 1) fixed for *A, C*, and *S*, and the second five (nearest source 2) fixed for *a, c*, and *s*. This simulates secondary contact. Each time step began with symmetrical dispersal between neighboring demes, followed by random mating within demes. In one set of simulations, I followed Dakin (2006) in assuming infinite source populations unaffected by gene flow (remaining fixed for their respective alleles for all time). In a second set, I allowed gene flow into the source populations, assuming they received half the number of immigrants (because they have only one rather than two neighbors). I report the disequilibria among loci in the adults (after dispersal, before mating); disequilibria in the offspring are lower in magnitude (by factors of *v*, $\frac{v}{2}$, and $\frac{1}{2}$) but the differences between cyto-symbiont, nuclear-symbiont, and cyto-nuclear associations are greater.

I iterated the system of equations to illustrate transient and equilibrium patterns using the R code in Online Appendix B. To check the effects of genetic drift on these predictions, I also modeled a system of finite populations. At each generation in each deme, I sampled *N* adults from the focal deme with probability 1 − *m* and from each neighboring deme with probability *m*/2. Then I sampled *N* offspring from the frequency distribution described by Equation (9) given the adult genotype frequencies.

(9) $$\begin{array}{l} {X^{\prime }}_{CSAA}=P_{A}\left[v\left(X_{\text{CSAA}}+\frac{1}{2}X_{\text{CSAa}}\right)+\left(1-v\right)P_{S}\left(X_{\text{CSAA}}+\frac{1}{2}X_{\text{CSAa}}+X_{\text{CsAA}}+\frac{1}{2}X_{\text{CsAa}}\right)\right]\\ {X^{\prime }}_{\text{CSAa}}=v\left(P_{A}X_{\text{CSaa}}+P_{a}X_{\text{CSAA}}+\frac{1}{2}X_{\text{CSAa}}\right)+\\ \;\left(1-v\right)P_{S}\left(P_{A}X_{\text{CSaa}}+P_{a}X_{\text{CSAA}}+\frac{1}{2}X_{\text{CSAa}}+P_{A}X_{\text{Csaa}}+P_{a}X_{\text{CsAA}}+\frac{1}{2}X_{\text{CsAa}}\right)\\ {X^{\prime }}_{\text{CSaa}}=P_{a}\left[v\left(X_{\text{CSaa}}+\frac{1}{2}X_{\text{CSAa}}\right)+\left(1-v\right)P_{S}\left(X_{\text{CSaa}}+\frac{1}{2}X_{\text{CSAa}}+X_{\text{Csaa}}+\frac{1}{2}X_{\text{CsAa}}\right)\right]\\ {X^{\prime }}_{\text{CsAA}}=P_{A}\left[v\left(X_{\text{CsAA}}+\frac{1}{2}X_{\text{CsAa}}\right)+\left(1-v\right)P_{s}\left(X_{\text{CSAA}}+\frac{1}{2}X_{\text{CSAa}}+X_{\text{CsAA}}+\frac{1}{2}X_{\text{CsAa}}\right)\right]\\ {X^{\prime }}_{\text{CsAa}}=v\left(P_{A}X_{\text{Csaa}}+P_{a}X_{\text{CsAA}}+\frac{1}{2}X_{\text{CsAa}}\right)+\\ \;\left(1-v\right)P_{s}\left(P_{A}X_{\text{CSaa}}+P_{a}X_{\text{CSAA}}+\frac{1}{2}X_{\text{CSAa}}+P_{A}X_{\text{Csaa}}+P_{a}X_{\text{CsAA}}+\frac{1}{2}X_{\text{CsAa}}\right)\\ {X^{\prime }}_{\text{Csaa}}=P_{a}\left[v\left(X_{\text{Csaa}}+\frac{1}{2}X_{\text{CsAa}}\right)+\left(1-v\right)P_{s}\left(X_{\text{CSaa}}+\frac{1}{2}X_{\text{CSAa}}+X_{\text{Csaa}}+\frac{1}{2}X_{\text{CsAa}}\right)\right]\\ {X^{\prime }}_{\text{cSAA}}=P_{A}\left[v\left(X_{\text{cSAA}}+\frac{1}{2}X_{\text{cSAa}}\right)+\left(1-v\right)P_{S}\left(X_{\text{cSAA}}+\frac{1}{2}X_{\text{cSAa}}+X_{\text{csAA}}+\frac{1}{2}X_{\text{csAa}}\right)\right]\\ {X^{\prime }}_{\text{cSAa}}=v\left(P_{A}X_{\text{cSaa}}+P_{a}X_{\text{cSAA}}+\frac{1}{2}X_{\text{cSAa}}\right)+\\ \;\left(1-v\right)P_{S}\left(P_{A}X_{\text{cSaa}}+P_{a}X_{\text{cSAA}}+\frac{1}{2}X_{\text{cSAa}}+P_{A}X_{\text{csaa}}+P_{a}X_{\text{csAA}}+\frac{1}{2}X_{\text{csAa}}\right)\\ {X^{\prime }}_{\text{cSaa}}=P_{a}\left[v\left(X_{\text{cSaa}}+\frac{1}{2}X_{\text{cSAa}}\right)+\left(1-v\right)P_{S}\left(X_{\text{cSaa}}+\frac{1}{2}X_{\text{cSAa}}+X_{\text{csaa}}+\frac{1}{2}X_{\text{csAa}}\right)\right]\\ {X^{\prime }}_{\text{csAA}}=P_{A}\left[v\left(X_{\text{csAA}}+\frac{1}{2}X_{\text{csAa}}\right)+\left(1-v\right)P_{s}\left(X_{\text{cSAA}}+\frac{1}{2}X_{\text{cSAa}}+X_{\text{csAA}}+\frac{1}{2}X_{\text{csAa}}\right)\right]\\ {X^{\prime }}_{\text{csAa}}=v\left(P_{A}X_{\text{csaa}}+P_{a}X_{\text{csAA}}+\frac{1}{2}X_{\text{csAa}}\right)+\\ \;\left(1-v\right)P_{s}\left(P_{A}X_{\text{cSaa}}+P_{a}X_{\text{cSAA}}+\frac{1}{2}X_{\text{cSAa}}+P_{A}X_{\text{csaa}}+P_{a}X_{\text{csAA}}+\frac{1}{2}X_{\text{csAa}}\right)\\ {X^{\prime }}_{\text{csaa}}=P_{a}\left[v\left(X_{\text{csaa}}+\frac{1}{2}X_{\text{csAa}}\right)+\left(1-v\right)P_{s}\left(X_{\text{cSaa}}+\frac{1}{2}X_{\text{cSAa}}+X_{\text{csaa}}+\frac{1}{2}X_{\text{csAa}}\right)\right] \end{array}$$

#### Epistasis and gene-culture coevolution: cattle as symbionts

I implemented the Feldman and Cavalli-Sforza (FCS) model as follows (Feldman and Cavalli-Sforza, 1989). The first step is mating and mother-offspring transmission of the symbiote with probability *v* (*U* vs. *u* represent presence or absence of the symbiote), such that the offspring gene-culture distribution is

(10) $$\begin{array}{l} {X^{\prime }}_{\text{CUAA}}=vp_{A}\text{​}\left(X_{\text{CUAA}}+\frac{1}{2}X_{\text{CUAa}}\right)\\ {X^{\prime }}_{\text{CUAa}}=v(p_{A}X_{\text{CUaa}}+p_{a}X_{\text{CUAA}}+\frac{1}{2}X_{\text{CUAa}})\\ {X^{\prime }}_{\text{CUaa}}=vp_{a}\text{​}\left(X_{\text{CUaa}}+\frac{1}{2}X_{\text{CUAa}}\right)\\ {X^{\prime }}_{\text{CuAA}}=p_{A}\text{​}\left(X_{\text{CuAA}}+\frac{1}{2}X_{CuAa}\right)+\left(1-v\right)p_{A}\text{​}\left(X_{\text{CUAA}}+\frac{1}{2}X_{\text{CUAa}}\right)\\ {X^{\prime }}_{\text{CuAa}}=p_{A}X_{\text{Cuaa}}+p_{a}X_{\text{CuAA}}+\frac{1}{2}X_{\text{CuAa}}+\left(1-v\right)\left(p_{A}X_{\text{CUaa}}+p_{a}X_{\text{CUAA}}+\frac{1}{2}X_{\text{CUAa}}\right)\\ {X^{\prime }}_{\text{Cuaa}}=p_{a}\text{​}\left(X_{\text{Cuaa}}+\frac{1}{2}X_{\text{CuAa}}\right)+\left(1-v\right)p_{a}\text{​}\left(X_{\text{CUaa}}+\frac{1}{2}X_{\text{CUAa}}\right)\\ {X^{\prime }}_{\text{cUAA}}=vp_{A}\text{​}\left(X_{\text{cUAA}}+\frac{1}{2}X_{\text{cUAa}}\right)\\ {X^{\prime }}_{\text{cUAa}}=v\text{​}\left(p_{A}X_{\text{cUaa}}+p_{a}X_{\text{cUAA}}+\frac{1}{2}X_{\text{cUAa}}\right)\\ {X^{\prime }}_{\text{cUaa}}=vp_{a}\text{​}\left(X_{\text{cUaa}}+\frac{1}{2}X_{\text{cUAa}}\right)\\ {X^{\prime }}_{\text{cuAA}}=p_{A}\text{​}\left(X_{\text{cuAA}}+\frac{1}{2}X_{\text{cuAa}}\right)+\left(1-v\right)p_{A}\text{​}\left(X_{\text{cUAA}}+\frac{1}{2}X_{\text{cUAa}}\right)\\ {X^{\prime }}_{\text{cuAa}}=p_{A}X_{\text{cuaa}}+p_{a}X_{\text{cuAA}}+\frac{1}{2}X_{\text{cuAa}}+\left(1-v\right)\left(p_{A}X_{\text{cUaa}}+p_{a}X_{\text{cUAA}}+\frac{1}{2}X_{\text{cUAa}}\right)\\ {X^{\prime }}_{\text{cuaa}}=p_{a}\text{​}\left(X_{\text{cuaa}}+\frac{1}{2}X_{\text{cuAa}}\right)+\left(1-v\right)p_{a}\text{​}\left(X_{\text{cUaa}}+\frac{1}{2}X_{\text{cUAa}}\right) \end{array}$$

where *p*_*A*_ and *p*_*a*_ are the host allele frequencies. In the second step, following Feldman and Cavalli-Sforza (1989), offspring have a chance of picking up the symbiote via horizontal transmission. That is, each individual with trait *u* has probability *fp*_*U*_ of being converted to trait *U* (*p*_*U*_ is the frequency of the symbiote *U* among other hosts in the population). The third step is selection according to

(11) $$\begin{array}{l} W_{.\text{UAA}}=1+s_{1}\;W_{.\text{uAA}}=1\\ W_{.\text{UAa}}=1+s_{1}\;W_{.\text{uAa}}=1\\ W_{.\text{Uaa}}=1-s_{2}\;W_{.\text{uaa}}=1 \end{array}$$

R code implementing the FCS model is provided in Online Appendix C.

#### Hitchhiking effects

R code for illustrating hitchhiking effects is provided in Online Appendices D and E. These give examples of commands for iterating the FCS model in a series of stepping stones after secondary contact and keeping track of allele and symbiont frequencies.
